# Surgical removal of an ectopic haemodialysis catheter in the brachiocephalic artery: a case report

**DOI:** 10.1093/jscr/rjae329

**Published:** 2024-05-24

**Authors:** Isqandar Adnan, Nurul Hafizah Zailani, Vimal Varma, Mardhiah Sarah Harnani Mansor, Raja Amin Raja Mokhtar, Muhammad 'Abid Amir

**Affiliations:** Department of Anaesthesiology and Intensive Care, Faculty of Medicine, Universiti Teknologi MARA, Sungai Buloh Campus, Sungai Buloh 47000, Selangor, Malaysia; Department of Cardiovascular and Thoracic Surgery, Faculty of Medicine, Universiti Teknologi MARA, Sungai Buloh Campus, Jalan Hospital, Sungai Buloh 47000, Selangor, Malaysia; Department of Anaesthesiology and Intensive Care, Faculty of Medicine, Universiti Teknologi MARA, Sungai Buloh Campus, Sungai Buloh 47000, Selangor, Malaysia; Department of Anaesthesiology and Intensive Care, Faculty of Medicine, Universiti Teknologi MARA, Sungai Buloh Campus, Sungai Buloh 47000, Selangor, Malaysia; Department of Cardiovascular and Thoracic Surgery, Faculty of Medicine, Universiti Teknologi MARA, Sungai Buloh Campus, Jalan Hospital, Sungai Buloh 47000, Selangor, Malaysia; Department of Cardiovascular and Thoracic Surgery, Faculty of Medicine, Universiti Teknologi MARA, Sungai Buloh Campus, Jalan Hospital, Sungai Buloh 47000, Selangor, Malaysia

**Keywords:** central venous catheter, inadvertent artery cannulation

## Abstract

Percutaneous central vein catheterization is commonly performed to access venous circulation for various clinical indications. However, unintentional arterial puncture may occur which can result in catastrophic complications. We report a case of an inadvertent right brachiocephalic artery cannulation in a 77-year-old lady planned for haemodialysis via a percutaneous internal jugular vein vascular access performed under ultrasound guidance. As an intravascular stent is not favourable in view of the close proximity of the right common carotid artery to the site of puncture as well as the risk of massive bleeding upon simple removal of the catheter, an open surgical removal via a median sternotomy was performed. Acquiring the competency in procedural skills, an understanding of the surgical anatomy and anticipating impending complications are of paramount importance in preventing as well as in mitigating the above complication.

## Introduction

Central venous catheters are commonly inserted for the administration of medications, fluids, nutrition and as a vascular access for haemodialysis. Current guidelines recommend central venous catheter insertion under ultrasound guidance to avoid iatrogenic injury to nearby structures [1]. Post-procedural chest radiograph is obtained to confirm the position of the catheter. The rate of arterial cannulation during internal jugular vein catheterization can be as high as 6.3–9.4% [2]. Majority of the incidents involve subclavian and common carotid artery. Here we report a case of a right internal jugular catheter traversing the right brachiocephalic artery ending above the aortic valve.

## Case report

**Figure 1:**
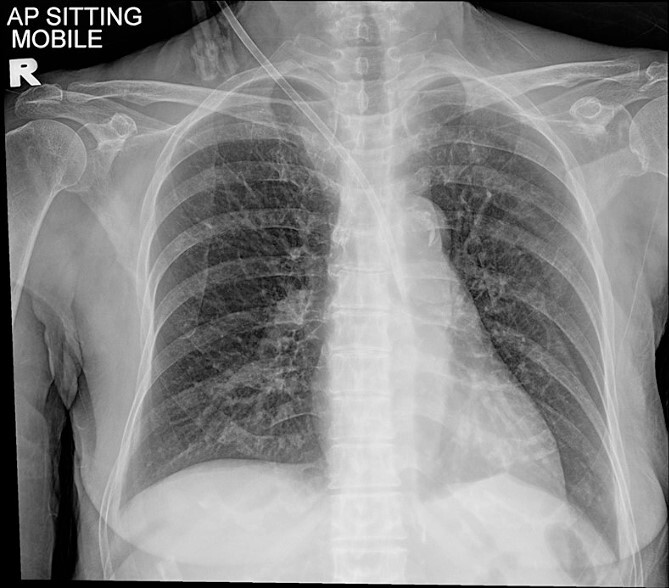
Chest radiograph showing a malpositioned central vein catheter.

**Figure 2:**
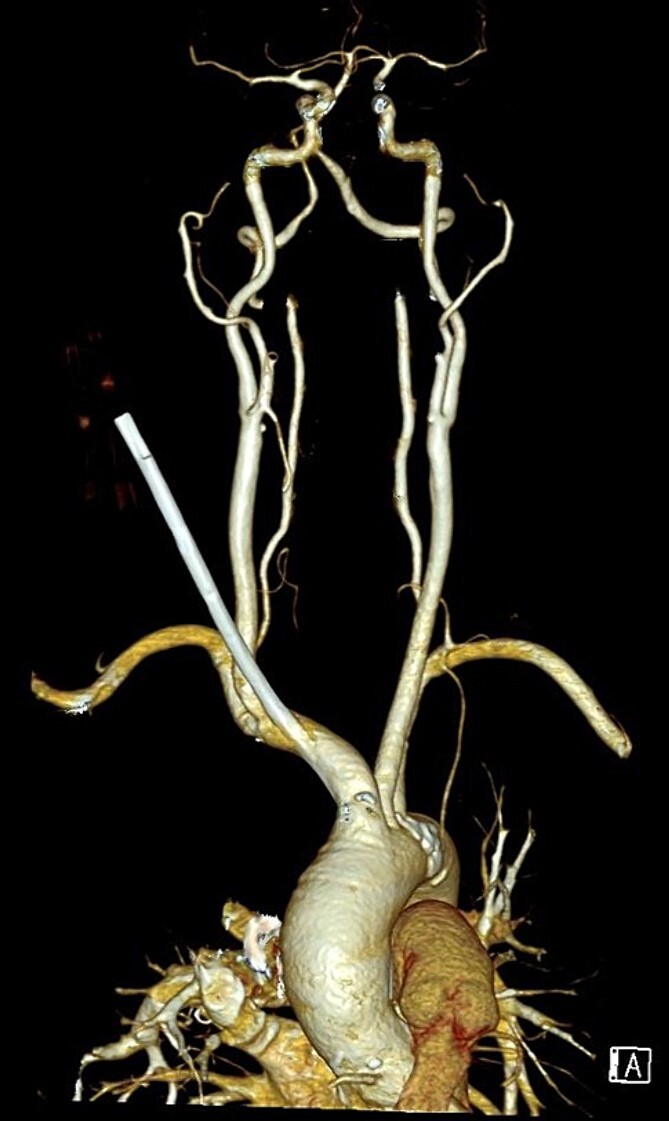
3-dimensional reconstruction of a CT angiogram of the neck and thorax depicting the ectopic haemodialysis catheter in the brachiocephalic artery.

A 77-year-old lady with underlying type 2 diabetes mellitus, hypertension, and newly diagnosed end-stage renal failure presented with a 1-week history of vomiting, diarrhoea, and loss of appetite. Clinically she was uraemic necessitating haemodialysis. A vascular access was attempted using a 15 cm, 12 Fr haemodialysis catheter through her right internal jugular vein under ultrasound guidance. However, a chest radiograph post procedure showed a malpositioned catheter (Fig. 1). A computed tomography angiogram of the neck and thorax with 3-dimensional image reconstruction revealed a through-and-through right internal jugular vein puncture resulting in an inadvertent right brachiocephalic artery cannulation. The puncture point was identified near the bifurcation of the right brachiocephalic artery with the tip lying just superior to the aortic valve (Fig. 2). She was urgently referred to the cardiothoracic centre for further management.

**Figure 3:**
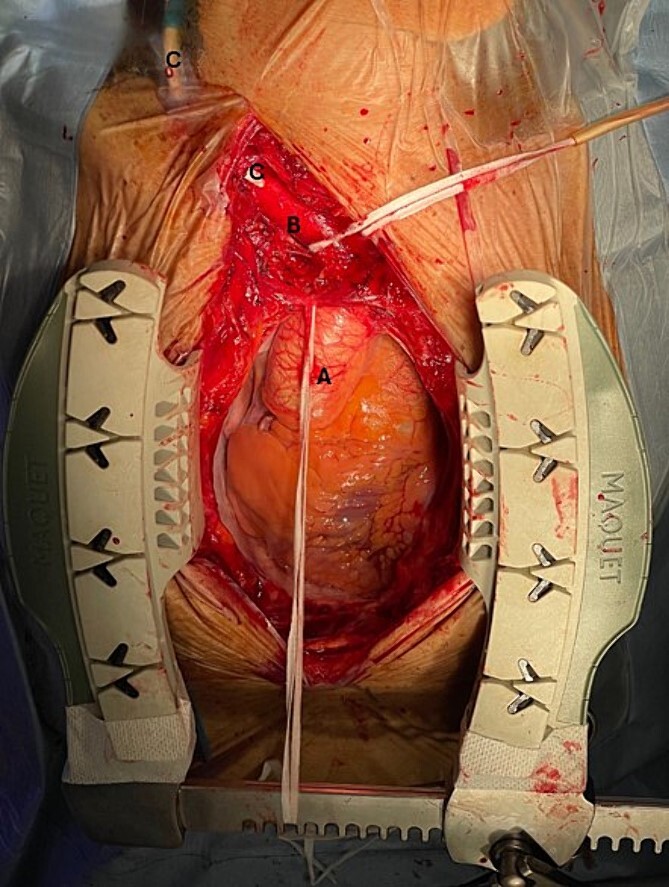
Intraoperative findings: A: Ascending aorta, B: Brachiocephalic artery, C: Malpositioned haemodialysis catheter in the brachiocephalic artery.

In view of the puncture site which was near the take-off of the right common carotid artery, an intravascular stent placement upon extraction of the catheter was not possible. A simple removal of the catheter with direct compression was similarly not feasible due to the overlying sternal bone, which would have led to massive haemmorhage. As such, a surgical removal via median sternotomy with direct suture repair was undertaken (Fig. 3). The catheter was removed in a single attempt with external compression to the neck at the skin puncture site. The arterial puncture site was repaired immediately post-removal. There were no immediate complications and the patient was extubated well.

## Discussion

A central venous catheter serves many purposes. These include infusion of fluids, medications, and nutrition. Its use can be extended as a vascular access for haemodialysis, to initiate extracorporeal therapy such as extracorporeal membrane oxygenator, transvenous instrumentation such as transvenous cardiac pacing, and placement of inferior vena cava filter.

The internal jugular vein is among the most common central venous access owing to its superficial and convenient location. The anatomical space located between the medial and lateral heads of the sternocleidomastoid muscle serves as a crucial landmark for blind insertion puncture. However, it is essential to note that this method has a failure rate ranging from 7.0% to 19.4% [3].

The real-time ultrasound guidance method is recommended with lesser complication compared to the anatomical landmark method [4]. Higher frequency (7 MHz) probe is preferred as it offers better visualization of superficial and nearby structures. However, despite the utilization of ultrasound, inadvertent arterial cannulation is still inevitable [5]. This is either due to misalignment between the needle and the imaging screen or due to through-and-through venous puncture to the posteriorly positioned artery. The latter usually occurs because of poor needle control or over-compression of the vein by the ultrasound probe. It is imperative to reconfirm the position of the guidewire via short and long-axis view before proceeding with dilation and advancement of the catheter.

Various strategies have been described to manage catheter-related cervicothoracic arterial injury namely, (i) simple removal and direct compression, (ii) endovascular treatment, and (iii) surgical repair. Each method must be tailored to each patient considering the anatomy of the arterial puncture site and the patients’ haemodynamic stability. A systematic review by Dixon *et al.* [6] proposed successful treatment by removal and compression, endovascular methods, and open surgical repair were 5.6%, 94.6%, and 100%, respectively.

Although a more straightforward approach, the removal and direct compression technique is associated with a 47% risk of developing major complications requiring further intervention [7]. This option was not feasible in our case as the puncture site is not easily accessible owing to its location behind the sternum and sternoclavicular joint which would have made a direct compression ineffective.

Current endovascular interventions include balloon tamponade, coil embolization, an intravascular covered stent, and percutaneous closure device. In our case, utilizing either a stent or coiled embolization was not suitable due to the site of puncture which was in close proximity to the bifurcation of the brachiocephalic artery which may result in occlusion of the right common carotid artery. Percutaneous suture–mediated closure device is only indicated for common femoral artery and venous closure but its ‘off-label’ use has been successfully described in articles [8]. Nanda *et al*., 2021 [9] reported thromboembolic stroke following percutaneous closure of the right brachiocephalic artery. The calibre of the catheter used in our case is relatively large (12Fr) and would require multiple percutaneous mediated closure devices with a pre-close technique.

Although there have been numerous case reports suggesting the success of endovascular techniques in managing inadvertent arterial cannulation [10–12], not many are about managing brachiocephalic artery cannulation specifically.

Surgical exploration and vascular repair are usually preferred in patients with approachable puncture points and feasible anatomy. To date, there is no significant study exhibiting the superiority of endovascular over surgical approach. Surgery is also recommended for injury recognized >4 hours [6]. In this case, access was made through a median sternotomy with an extension of the incision to the right cranially for better exposure. Extra care must be taken to avoid injury to the right recurrent laryngeal nerve as it loops around the right subclavian artery laterally.

## Conclusion

This case is unique as inadvertent brachiocephalic artery cannulation is underreported in the literature. More studies and reports are needed to answer the conundrum regarding the best approach to this complication. Proficiency in carrying out procedural skills together with an astute knowledge in performing image-guided techniques, familiarity with surgical anatomy as well as anticipation of possible complications are important factors in preventing and in mitigating the above catastrophic complication.

## Data Availability

Due to privacy and confidentiality concerns related to patient medical records and information, we are unable to provide direct access to the raw patient data. However, we are open to addressing specific data-related inquiries or requests on a case-by-case basis. Researchers interested in obtaining specific data points or seeking clarification on our findings are encouraged to contact the corresponding author.
